# State Cannabis and Psychedelic Legislation and Microdosing Interest in the US

**DOI:** 10.1001/jamahealthforum.2024.1653

**Published:** 2024-06-28

**Authors:** Kevin H. Yang, Nora Satybaldiyeva, Matthew R. Allen, John W. Ayers, Eric C. Leas

**Affiliations:** 1 Department of Psychiatry, University of California San Diego School of Medicine, La Jolla; 2 Herbert Wertheim School of Public Health and Human Longevity Science, University of California San Diego, La Jolla; 3 University of California San Diego School of Medicine, La Jolla; 4 Qualcomm Institute, University of California San Diego, La Jolla; 5 Altman Clinical Translational Research Institute, University of California San Diego, La Jolla; 6 Division of Infectious Diseases and Global Public Health, Department of Medicine, University of California San Diego, La Jolla

## Abstract

**Importance:**

Despite growing interest in psychedelics, there is a lack of routine population-based surveillance of psychedelic microdosing (taking “subperceptual” doses of psychedelics, approximately one-twentieth to one-fifth of a full dose, over prolonged periods). Analyzing Google search queries can provide insights into public interest and help address this gap.

**Objective:**

To analyze trends in public interest in microdosing in the US through Google search queries and assess their association with cannabis and psychedelic legislative reforms.

**Design, Setting, and Participants:**

In this cross-sectional study, a dynamic event-time difference-in-difference time series analysis was used to assess the impact of cannabis and psychedelic legislation on microdosing search rates from January 1, 2010, to December 31, 2023. Google search rates mentioning “microdosing,” “micro dosing,” “microdose,” or “micro dose” within the US and across US states were measured in aggregate.

**Exposure:**

Enactment of (1) local psychedelic decriminalization laws; (2) legalization of psychedelic-assisted therapy and statewide psychedelic decriminalization; (3) statewide medical cannabis use laws; (4) statewide recreational cannabis use laws; and (5) all cannabis and psychedelic use restricted.

**Main Outcome and Measures:**

Microdosing searches per 10 million Google queries were measured, examining annual and monthly changes in search rates across the US, including frequency and nature of related searches.

**Results:**

Searches for microdosing in the US remained stable until 2014, then increased annually thereafter, with a cumulative increase by a factor of 13.4 from 2015 to 2023 (7.9 per 10 million to 105.6 per 10 million searches, respectively). In 2023, there were 3.0 million microdosing searches in the US. Analysis at the state level revealed that local psychedelic decriminalization laws were associated with an increase in search rates by 22.4 per 10 million (95% CI, 7.5-37.2), statewide psychedelic therapeutic legalization and decriminalization were associated with an increase in search rates by 28.9 per 10 million (95% CI, 16.5-41.2), statewide recreational cannabis laws were associated with an increase in search rates by 40.9 per 10 million (95% CI, 28.6-53.3), and statewide medical cannabis laws were associated with an increase in search rates by 11.5 per 10 million (95% CI, 6.0-16.9). From August through December 2023, 27.0% of the variation in monthly microdosing search rates between states was explained by differences in cannabis and psychedelics legal status.

**Conclusion and Relevance:**

This cross-sectional study found that state-led legislative reforms on cannabis and psychedelics were associated with increased public interest in microdosing psychedelics.

## Key Points

**Question:**

How has public interest in microdosing in the US changed over the past decade, and what impact have legislative changes related to psychedelics and cannabis had on this interest?

**Findings:**

In this cross-sectional study, an analysis of Google Trends data from January 2010 to December 2023 revealed a notable increase in searches for microdosing across the US. This uptrend was associated with legislative changes concerning both psychedelics and cannabis, as indicated by a dynamic event-time difference-in-difference time series analysis.

**Meaning:**

Interest in microdosing is increasing across the US and is influenced by legislative reforms concerning both psychedelics and cannabis.

## Teaser

This cross-sectional study analyzes trends in public interest in microdosing in the US through Google search queries and assesses their association with cannabis and psychedelic legislative reforms.

## Introduction

In recent years, the practice of microdosing has gained cultural significance and presents new public health risks. Microdosing entails taking subperceptual doses of psychedelics, approximately one-twentieth to one-fifth of a full dose, over prolonged periods, as a wellness treatment to improve cognition, mood, and overall health without the intense psychotropic effects of larger doses.^1,2,3,4,5,6,7^ Common microdosed substances include lysergic acid diethylamide (LSD), psilocybin, and 3,4-methylenedioxymethamphetamine (MDMA).^1,4^ However, clinical evidence on microdosing is lacking, and data on its benefits are contradictory, suggesting benefits may be attributable to placebo effects.^1,8,9,10^ The practice also raises safety concerns (eg, headaches, insomnia, anxiety, paranoia, and theoretical risks of valvular heart disease) due to adverse effects, self-medication, inaccurate dosing, and the use of illicit controlled substances.^2,4,5,6,11,12,13^ Despite the health implications, the extent of public interest in microdosing and its primary drivers remain largely unknown. In addition, endorsements from public figures and media coverage of microdosing may contribute to increased public interest.^14,15^ Consequently, there is a critical need for research establishing population benchmark measures of public interest in microdosing psychedelics.

Significant shifts in policy on psychedelics in the US could have increased public interest in microdosing. An era called the *psychedelic renaissance* started around 2010 and has been marked by growth in clinical research and legislative reforms.^16,17^ During this era, the US Food and Drug Administration (FDA) granted breakthrough therapy designations to MDMA for posttraumatic stress disorder (PTSD) in 2017 and to psilocybin for treatment-resistant depression (TRD) in 2018 and major depressive disorder (MDD) in 2019.^18,19^ In addition, the FDA granted formal approval to esketamine for the treatment of TRD in 2019.^20^ Alongside federal reform, a wave of state and local policy reforms signify a shift toward acceptance of psychedelics, with projections of widespread decriminalization of psychedelics by 2037.^18,21,22,23^

State-led cannabis use policy reforms in the past decade could have also impacted public interest in microdosing. These reforms have led to the establishment of legal markets for cannabis in many US states, making psychoactive cannabis products legally accessible to most US adults.^24^ This expanded marketplace for psychoactive products could increase public experimentation with psychedelics, including microdosing, as drug manufacturers, including those previously dedicated to cannabis, have already begun expanding their product lines to incorporate psychedelic substances.^25,26^ In addition, state-led legalization of medical cannabis may indicate greater openness to alternative therapies such as psilocybin for depression.

Herein, we explore public interest in microdosing using aggregate Google search trends and examine its relationship with cannabis and psychedelic legislative reforms. Although nationally representative surveys such as the National Survey on Drug Use and Health and Monitoring the Future have shown increases in self-reported psychedelic use in recent years, they fall short of detailing dosing specifics.^27,28,29^ Google searches can serve as a measure of demand or interest in specific products,^30^ including psychoactive substances,^31,32,33^ with search content potentially revealing search intent. We used trends in Google searches for “microdosing” to describe public interest across the US and a difference-in-differences (DiD) approach to test whether public interest increased following the enactment of statewide medical and recreational cannabis use policies and state or local psychedelic use policies.^34^ We also report the search queries most commonly related to microdosing as an assessment of interest in substance use.

## Methods

### Study Data and Measures

#### Google Trends

The University of California, San Diego institutional review board exempted the analyses from review due to deidentified, publicly available data. The study followed Strengthening the Reporting of Observational Studies in Epidemiology (STROBE) reporting guidelines.

We measured aggregate Google search rates mentioning “microdosing,” “micro dosing,” “microdose,” or “micro dose” within the US and across US states from January 1, 2010, through December 31, 2023. Search rates were measured as the fraction of total Google searches and expressed per 10 million. Trends were obtained from the Google API Client library in Python.^35^

#### Related Google Queries

Related queries are terms that Google users who searched for any of the microdosing terms also searched for in the same browser session, which may reveal the potential intent of the microdosing search. Related terms were scored on a relative scale where a value of 100 is the most commonly searched query in the time period and location, 50 is a query searched half as often as the top related query, and so on. We obtained the top 25 related terms for the US from the Google trends dashboard^36^ using the same microdosing keywords for each year from 2010 through 2023.

#### Medical and Recreational Cannabis Policy Enactment

State medical and recreational cannabis use policies were obtained from DISA Global Solutions.^37^ Each state was categorized as “legal” or “not legal.” For the former, we recorded the date of policy enactment (eTable 1 in Supplement 1).

#### Psychedelic Policy Enactment

We developed an up-to-date table of jurisdictions where entheogenic plants or psilocybin have been legalized or decriminalized based on public sources.^18,22,38,39^ To verify and determine enactment dates, 2 authors (K.Y. and N.S.) searched city and state council websites for official documentation. When resolutions were not on official websites, the authors searched Ballotpedia.org^40^ and local news articles. Entheogenic plant definitions often included the full spectrum of psychedelic plants, fungi, and natural materials containing indole amines, tryptamines, and phenethylamines, including psilocybin mushrooms, ayahuasca, cacti, and iboga. Although the definitions slightly differed by jurisdiction, all laws included psilocybin along with at least 1 other plant-derived psychedelic. Jurisdictions were grouped by state, and states were categorized according to whether they had (1) any psychedelic decriminalization laws in their jurisdiction, meaning cities or counties in the state that have decriminalized or deprioritized criminal penalties associated with producing, possessing, or consuming some psychedelics; or (2) legalization of psychedelic-assisted therapy and statewide psychedelic decriminalization (all state categorizations and policy sources are in eTable 2 in Supplement 1).

### Statistical Analyses

#### Policy Adoption Curves

Growth in policy adoption was described for each of the 4 policy types: (1) statewide medical cannabis use laws; (2) statewide recreational cannabis use laws, (3) local psychedelic decriminalization laws, and (4) legalization of psychedelic-assisted therapy and statewide psychedelic decriminalization laws. These were described as the total number of policy enactments at the end of 2023.

#### Trends in Google Search Rates

We calculated the annualized change in microdosing search rates nationally and for each US state, summarizing them using means and ranges.

#### Absolute Search Volume Estimate

We estimated the approximate number of microdosing searches for the US by multiplying the rate of microdosing searches in the US in 2023 by an estimate for the total number of searches in the US in 2023. First, we obtained the monthly search total originating from US desktop computers for the month of July 2023 from Comscore.^41^ We assumed this monthly desktop search total was constant over the 12 months in 2023. Second, we adjusted this number by the fraction of searches originating from desktop computers in the US (38.1%) estimated by the search engine optimization company SISTRIX using their proprietary database.^42^ With these numbers, we estimated the total number of searches during this period using the following equation: QF × CS × N × AF; where QF is the fraction of all queries that are microdosing searches (expressed per 1 search) in the US in 2023, CS is the Comscore estimate of total monthly desktop search volume in 2023, N is the number of months in the study period, which in this case was 12 months, and AF is the adjustment factor for the estimated proportion of Google searches that originated from desktop computers.

#### Difference-in-Differences Analyses

Because the adoption of the state or local psychedelic use policies was staggered throughout the study period rather than implemented at once, we relied on the dynamic event DiD framework proposed by Callaway and Sant’Anna.^43^ We conducted separate analyses for each of the cannabis and psychedelic policies of interest. One assumption of DiD is that the trends in the outcome are parallel between the control and the treatment in the periods prior to treatment.^34^ We tested this assumption by assessing whether the 95% CIs for all pretreatment effect estimates included zero. Once this assumption was assessed, the quantities of interest were group-time average treatment effects, with pooling based on the event-study aggregation across all posttreatment periods. For these quantities, we considered the year before the policy enactment to be the reference year and the control group to be states that never adopted the policy. In sensitivity analyses, we also varied the treatment year to be 1 year before or after enactment to assess the potential for lead-in or delayed associations (eTable 3 in Supplement 1), but the results were similar (all 95% CIs were overlapping).

#### Cross-Sectional Analysis of Google Search Rates Across Policy Groups

We described the rate of microdosing Google searches cross-sectionally across the state cannabis and psychedelic use policies for the final months in which all enactments had been made (August 2023 through December 2023) using means and analysis of variance.

#### Analysis of Related Google Searches

We generated a heat map to assess the evolution of top Google search queries related to microdosing, ordering terms by their average search frequency value since 2015. All analyses were conducted using R statistical software (version 4.3.1; R Foundation).

## Results

**Figure 1.**
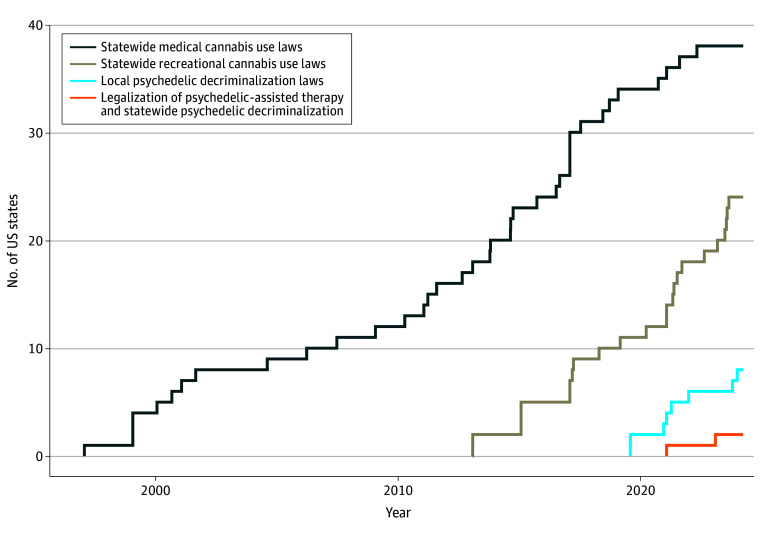
Cumulative Frequency of Policy Changes by US State Local psychedelic decriminalization laws means there are cities or counties in the state that have decriminalized psychedelics. Categorization of jurisdictions with medical cannabis use laws can be found in eTable 1 in Supplement 1. Categorization of jurisdictions with recreational cannabis use laws can be found in eTable 1 in Supplement 1. Categorization of jurisdictions with psychedelic decriminalization and assisted-therapy laws can be found in eTable 2 in Supplement 1.

Figure 1 presents the timeline of the diffusion of state-led cannabis and psychedelic policies across the US. As of December 31, 2023, 38 US states (including the District of Columbia) had adopted medical cannabis use policies, 24 (including the District of Columbia) had adopted recreational cannabis use policies, 8 (including the District of Columbia) had a local jurisdiction that decriminalized psychedelics, and 2 had legalized psychedelic-assisted therapy and decriminalized psychedelics statewide.

**Figure 2.**
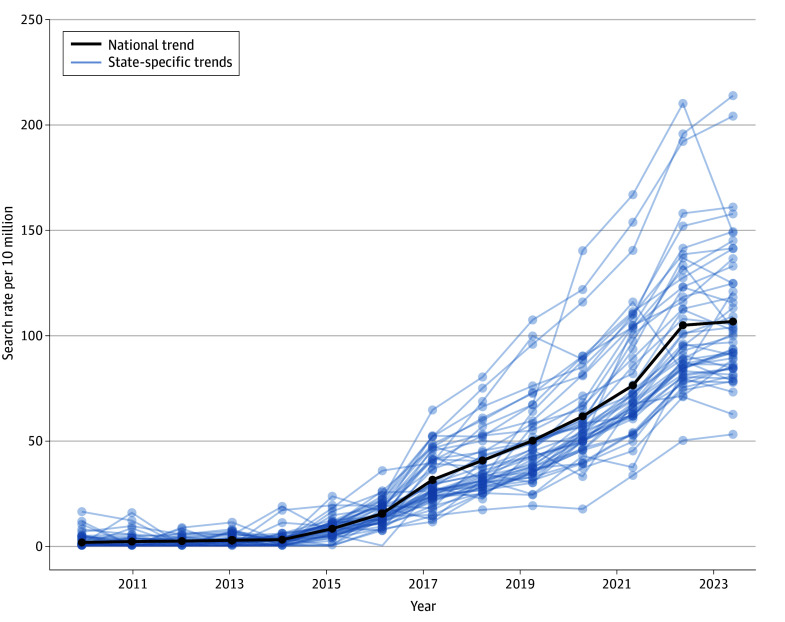
Google Searches for Microdosing Within and Across US States From 2010 to 2023 Excludes North Dakota, South Dakota, and Wyoming, which had unreliable estimates throughout the period.

Nationally, microdosing searches were stable from 2010 through 2014 but then increased each subsequent year thereafter (Figure 2). During this period, searches increased by an average of 12.2 per 10 million per year and cumulatively increased by a factor of 13.4 from 7.9 per 10 million in 2015 to 105.6 per 10 million in 2023. In absolute terms, there were 3.0 million microdosing Google searches in the US in 2023. The states with the fastest growth rates were Colorado (25.6 per 10 million per year), Oregon (23.9 per 10 million per year), and Washington (18.7 per 10 million per year). Consequently, by 2023, microdosing searches were highest in Oregon (202.5 per 10 million), Colorado (159.6 per 10 million), and Washington (159.6 per 10 million), and varied considerably across US states.

**Figure 3.**
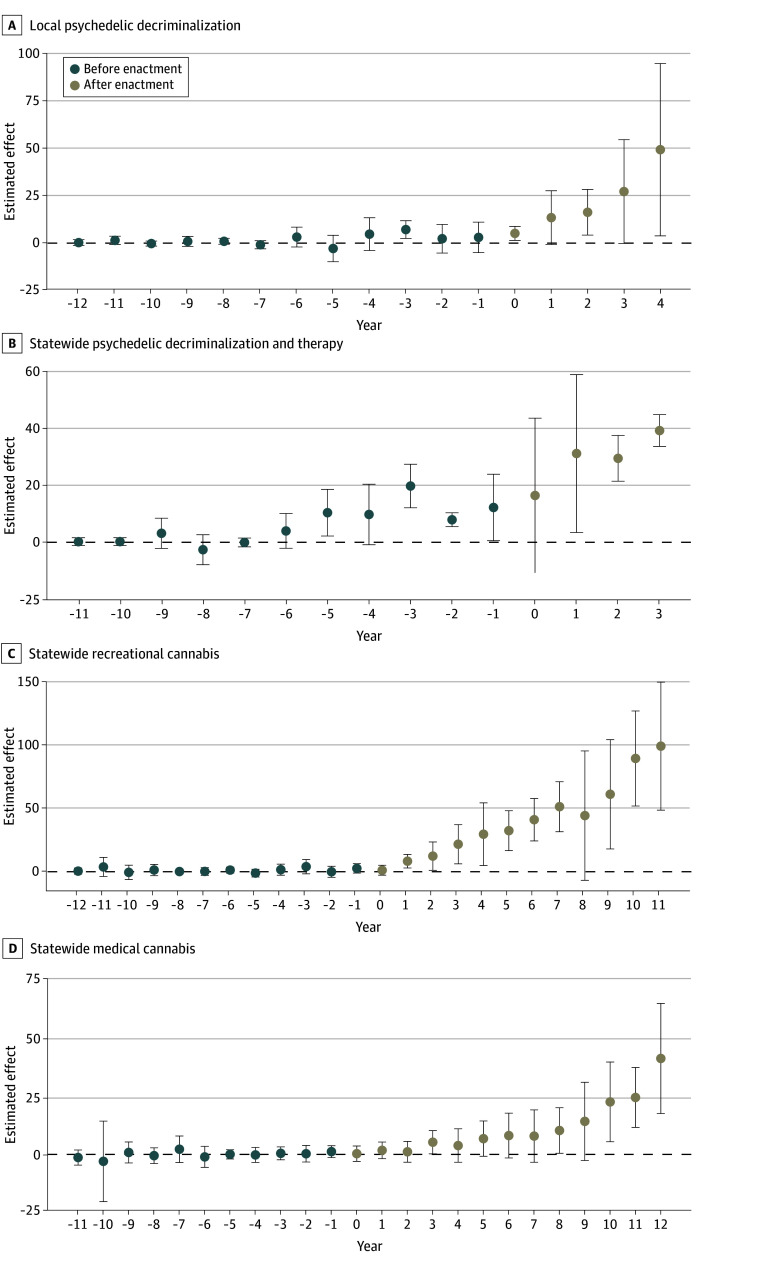
Associations Between Cannabis and Psychedelic Policy and Trends in Google Searches for Microdosing. Local psychedelic decriminalization laws means there are cities or counties in the state that have decriminalized psychedelics. The whiskers indicate the 95% CIs. Year 0 is the year the policy was enacted. Categorization of jurisdictions with medical cannabis use laws can be found in eTable 1 in Supplement 1. Categorization of jurisdictions with recreational cannabis use laws can be found in eTable 1 in Supplement 1. Categorization of jurisdictions with psychedelic decriminalization and assisted-therapy laws can be found in eTable 2 in Supplement 1. Excludes North Dakota, South Dakota, and Wyoming, which had unreliable estimates throughout the period. Numeric values available in eTables 4-7 in Supplement 1.

Figure 3 presents the results of the DiD analysis testing the associations between state policies and microdosing interest (numeric values available in eTables 4-7 in Supplement 1). When analyzing pretreatment trends, we found no evidence of a differential microdosing trend between treated and control states (pretreatment 95% CIs crossed 0) for local psychedelic decriminalization laws (Figure 3A) or statewide recreational (Figure 3C) or medical (Figure 3D) cannabis use laws. However, there was minor evidence of a differential microdosing trend between treated and control states (a few pretreatment 95% CIs crossed 0) for statewide legalization of psychedelic-assisted therapy and decriminalization of psychedelics (Figure 3B).

Overall, enactment of local psychedelic decriminalization laws (Figure 3A) was associated with an increase in microdosing search rates by 22.4 per 10 million (95% CI, 7.5-37.2), enactment of statewide legalization of psychedelic-assisted therapy and decriminalization of psychedelics (Figure 3B) was associated with an increase in microdosing search rates by 28.9 per 10 million (95% CI, 16.5-41.2), and enactment of statewide recreational cannabis use laws (Figure 3C) was associated with an increase in microdosing search rates by 40.9 per 10 million (95% CI, 28.6-53.3). For each of these policies, microdosing search interest first significantly increased (95% CI did not include 0) the year of or 1-year following enactment. The enactment of statewide medical cannabis use laws was also associated with an increase in microdosing search rates by 11.5 per 10 million (95% CI, 6.0-16.9) for the period. However, the first year where searches were significantly elevated (95% CI did not include 0) was 8 years following enactment (Figure 3D).

**Figure 4.**
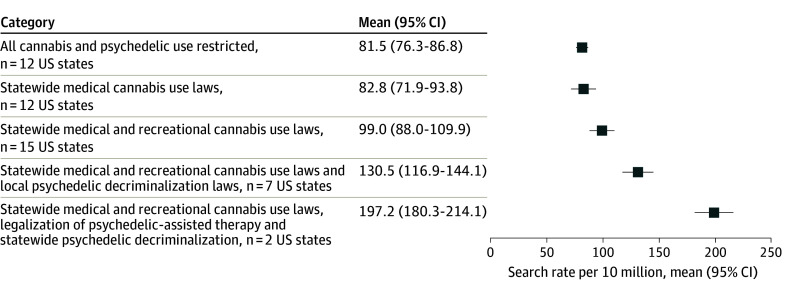
Associations Between Cannabis and Psychedelic Policies and Average Monthly Microdosing Search Rates From August to December 2023 Local psychedelic decriminalization laws means there are cities or counties in the state that have decriminalized psychedelics. August to December 2023 were selected to describe differences across policies, because these were the last months with no new policy enactments. Categorization of jurisdictions with medical cannabis use laws can be found in eTable 1 in Supplement 1. Categorization of jurisdictions with recreational cannabis use laws can be found in eTable 1 in Supplement 1. Categorization of jurisdictions with psychedelic decriminalization and assisted-therapy laws can be found in eTable 2 in Supplement 1. Excludes North Dakota, South Dakota, and Wyoming, which had unreliable estimates throughout the period.

By 2023, the legal status of cannabis and psychedelics explained 27.0% of the variance in monthly search rates (*R*^2^ = 0.27; *F* = 26.3; *P* < .001) (Figure 4**)**. Compared to states where all cannabis and psychedelic use was restricted (mean, 81.5 per 10 million; 95% CI, 76.3-86.8), monthly microdosing search rates were similar in states that had enacted medical cannabis use policies (mean, 82.8 per 10 million; 95% CI, 71.9-93.8). All other policies had a positive and dose-response association, such that interest in microdosing increased with increasingly permissive environments toward substance use. Compared to states where all cannabis and psychedelic use was restricted, monthly microdosing search rates were 1.2 times higher in states that had enacted both medical and recreational cannabis use policies (mean, 99.0 per 10 million; 95% CI, 88.0-109.9), 1.6 times higher in states that had enacted medical and recreational cannabis use policies and had local jurisdictions that decriminalized psychedelics (mean, 130.5 per 10 million; 95% CI, 116.9-144.1), and 2.4 times higher in states that had enacted medical and recreational cannabis use policies, had decriminalized psychedelics, and had legalized psychedelic-assisted therapy programs (mean, 197.2 per 10 million; 95% CI, 180.3-214.1).

**Figure 5.**
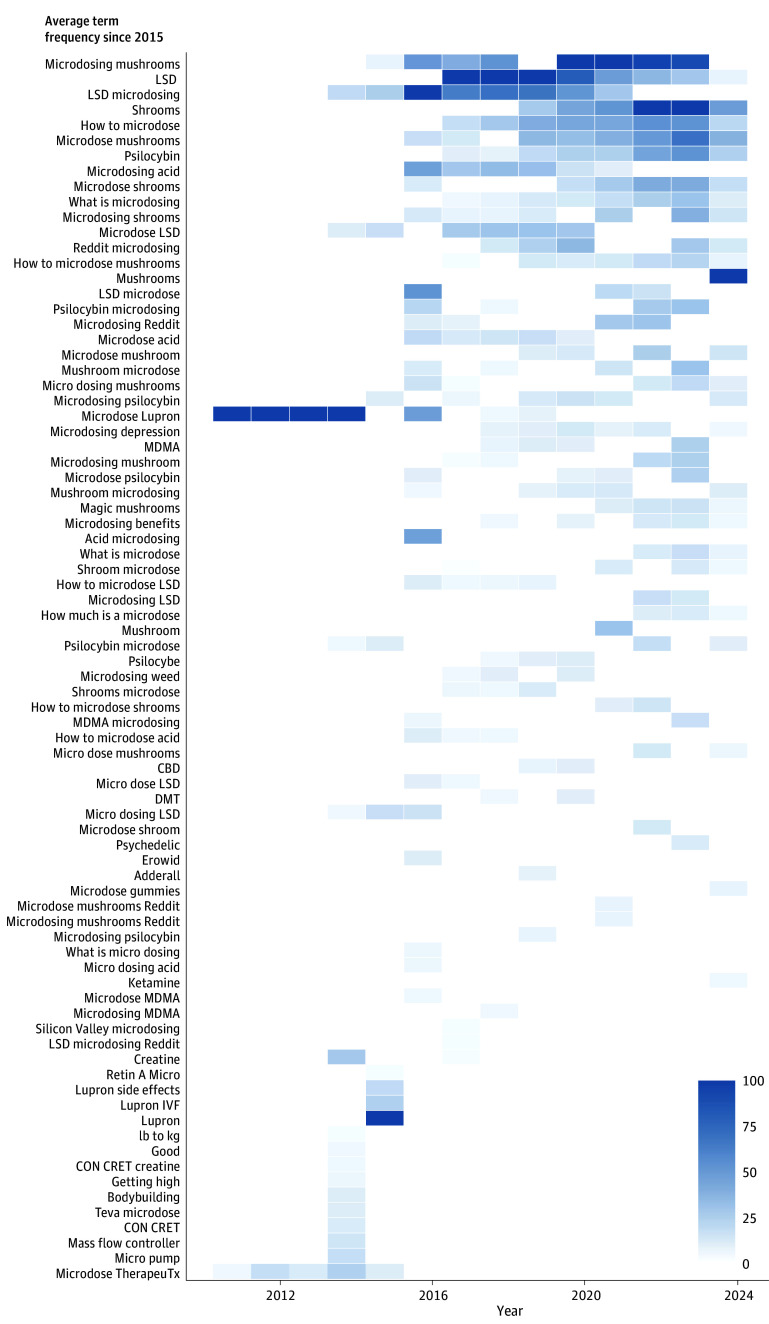
Trends in Related Terms Associated With Google Searches for Microdosing CBD indicates cannabidiol; DMT, N,N-Dimethyltryptamine; IVF, in vitro fertilization; LSD, lysergic acid diethylamide; MDMA, 3,4-methylenedioxymethamphetamine. The related queries are terms that Google users who searched for microdosing also searched for in the same browser session. Scoring is on a relative scale where a value of 100 is the most commonly searched query; 50 is a query searched half as often as the top related query, and so on.

Figure 5 illustrates the evolution of top Google search queries related to microdosing from 2010 to 2023. Before 2015, few of the top 25 terms were related to microdosing psychedelics. For example, the top related term from 2010 to 2014 was “microdose lupron,” which is a brand name for leuprolide acetate and is used for in vitro fertilization. After 2015, however, terms related to microdosing psychedelics predominated. For example, from 2015 to 2018, the top term related to microdosing searches was “LSD” and from 2019 through 2023, the top terms were “microdosing mushrooms,” “shrooms,” and “mushrooms.” Other keywords indicative of psychoactive drugs that also appeared in the top 25 related terms included “MDMA,” “ketamine,” “psilocybin,” and “weed.” Top-related terms also included queries seeking microdosing information (eg, “how to microdose,” “Reddit microdosing,” and “microdosing depression”).

## Discussion

Public interest in microdosing has grown in the US, particularly since 2015. Interest was highest in the 2 US states, Oregon and Colorado, that had enacted psychedelic-assisted therapy programs and decriminalized psychedelic use, but even implementation of local decriminalization of psychedelics and recreational cannabis use policies were associated with increased microdosing interest.

The observed associations of cannabis and psychedelic policy changes with increased interest in microdosing may be driven by several potential mechanisms. First, state-level medical and recreational cannabis legalization may have led to a broader acceptance of psychoactive substances and reduced stigma associated with their use, perhaps encouraging individuals to explore other psychoactive substances, such as psychedelics, for microdosing. Second, psychedelic decriminalization and regulatory approval of psychedelics for therapeutic use could enhance their public legitimacy, leading to increased interest in microdosing for recreational and mental health purposes. Third, the increasing prevalence of depression and suicidal ideation among adults, without any corresponding increases in mental health service utilization,^44,45^ may also influence individuals to explore microdosing for mental health purposes. These preliminary findings underscore the need for further research into the impact of legislative changes on microdosing practices.

Notably, laws pertaining to cannabis and psychedelics explained roughly a quarter of the variance in interest across US states, with greater interest in less restrictive states. An additional criterion for causality is temporality,^46^ and the dynamic event-time DiD analyses suggested that interest in microdosing increased following enactment of both cannabis and psychedelic laws. For psychedelics, the impact varied between local and statewide policies, possibly due to the timing of implementations, wider adoption of local jurisdiction laws compared to statewide policies, and differences in community characteristics affected by local vs state policies. For cannabis, the impact varied between medical and recreational policies, possibly due to greater impacts on public perceptions of cannabis and other psychoactive substances from recreational legalization, rather than medical legalization, which may be less accessible due to barriers (ie, physician prescription). As more states move toward legalization and decriminalization of psychedelics and cannabis, more research is needed to better understand the impact of such policies.

Prior to 2015, few of the top-related search terms were indicative of topics related to microdosing psychedelics (eg, lupron). Starting in 2015, however, search queries shifted toward psychedelic substances, with LSD initially leading until 2018. From 2019 onwards, searches for “mushrooms” and “shrooms” surpassed LSD, indicating a potential shift in interest toward different substances. Although we cannot confirm which mushroom species, the frequent mention of “psilocybin” points to a focused interest, which coincides with the breakthrough designation of psilocybin by the FDA for TRD in 2018 and MDD in 2019.^18^ Furthermore, the reliance on potentially unreliable information sources like Erowid^47^ and Reddit^48^ highlights the need for accessible evidence-based information to ensure consumers are well-informed about the benefits and risks of microdosing.^49^

### Limitations

Our findings should be interpreted in the context of several limitations. First, we studied Google search queries rather than direct usage given the lack of epidemiologic data on microdosing. However, in many other cases, searches have presaged increases in substance use and policy impacts,^32^ often years before confirmatory evidence becomes available.^50^ One indication that the potential interest in microdosing was indicative of interest in using substances was the frequency at which related queries mentioned specific psychedelics and how-to guides (eg, “how to microdose mushrooms”), but these findings should be validated and supplemented by other surveillance methods, such as surveys. Second, our analysis measured general interest in microdosing but did not differentiate between specific substances. Although related search terms such as psilocybin, LSD, and ketamine lend some credibility to the observed trends’ relevance to psychedelics, the relative prevalence of used substances for microdosing should be evaluated in future studies. Despite these limitations, our findings suggest that state-led legislative reforms, including both cannabis and psychedelic use reforms, are associated with increased public interest in microdosing psychedelics.

## Conclusions

The findings of this cross-sectional analysis suggest that rigorous clinical studies are needed to evaluate the safety profile and potential benefits of microdosing to inform evidence-based practices and policymaking to match public interest. Additional population-based surveillance is needed to identify who is microdosing, their reasons, and how these practices might change with the evolving legal landscape.

## Notes

Supplement 1. **eTable 1.** Categorization of states with medical or recreational cannabis use laws enacted as of January 2024 **eTable 2.** Categorization of jurisdictions with psychedelic laws enacted as of January 2024 **eTable 3**. Sensitivity analysis of cannabis and psychedelic policy effects on microdosing Google search trends changing the treatment period to 1 year prior to policy enactment **eTable 4.** Dynamic effects of local jurisdiction psychedelic decriminalization laws by event time and overall summary of ATTs based on event-study/dynamic aggregation for yearly data across 48 US states with reliable estimates of microdosing trends **eTable 5**. Dynamic effects of legalization of psychedelic-assisted therapy & statewide decriminalization laws by event time and overall summary of ATTs based on event-study/dynamic aggregation for yearly data across 48 US states with reliable estimates of microdosing trends **eTable 6. **Dynamic effects of statewide recreational cannabis use laws by event time and overall summary of ATTs based on event-study/dynamic aggregation for yearly data across 48 US states with reliable estimates of microdosing trends **eTable 7. **Dynamic effects of statewide medical cannabis use laws by event time and overall summary of ATTs based on event-study/dynamic aggregation for yearly data across 34 US states with reliable estimates of microdosing trends and no medical cannabis use laws as of 2010

Supplement 2. Data Sharing Statement
