# Supplementary material for: State Cannabis and Psychedelic Legislation and Microdosing Interest in the US
Source: JAMA Health Forum. 2024 Jun 28;5(6):e241653. doi: 10.1001/jamahealthforum.2024.1653 (PMC11214114; doi:10.1001/jamahealthforum.2024.1653)
Supplement: Supplement 1. — eTable 1. Categorization of states with medical or recreational cannabis use laws enacted as of January 2024 eTable 2. Categorization of jurisdictions with psychedelic laws enacted as of January 2024 eTable 3. Sensitivity analysis of cannabis and psychedelic policy effects on microdosing Google search trends changing the treatment period to 1 year prior to policy enactment eTable 4. Dynamic effects of local jurisdiction psychedelic decriminalization laws by event time and overall summary of ATTs based on event-study/dynamic aggregation for yearly data across 48 US states with reliable estimates of microdosing trends eTable 5. Dynamic effects of legalization of psychedelic-assisted therapy & statewide decriminalization laws by event time and overall summary of ATTs based on event-study/dynamic aggregation for yearly data across 48 US states with reliable estimates of microdosing trends eTable 6. Dynamic effects of statewide recreational cannabis use laws by event time and overall summary of ATTs based on event-study/dynamic aggregation for yearly data across 48 US states with reliable estimates of microdosing trends eTable 7. Dynamic effects of statewide medical cannabis use laws by event time and overall summary of ATTs based on event-study/dynamic aggregation for yearly data across 34 US states with reliable estimates of microdosing trends and no medical cannabis use laws as of 2010 [file jamahealthforum-e241653-s001.pdf]

Supplemental Online Content

Yang KH, Satybaldiyeva N, Allen MR, Ayers JW, Leas EC. State cannabis and psychedelic legislation and microdosing interest in the US. *JAMA Health Forum*. 2024;5(6):e241653. doi:10.1001/jamahealthforum.2024.1653

eTable 1. Categorization of states with medical or recreational cannabis use laws enacted as of January 2024

eTable 2. Categorization of jurisdictions with psychedelic laws enacted as of January 2024

eTable 3. Sensitivity analysis of cannabis and psychedelic policy effects on microdosing Google search trends changing the treatment period to 1 year prior to policy enactment

eTable 4. Dynamic effects of local jurisdiction psychedelic decriminalization laws by event time and overall summary of ATTs based on event-study/dynamic aggregation for yearly data across 48 US states with reliable estimates of microdosing trends

eTable 5. Dynamic effects of legalization of psychedelic-assisted therapy & statewide decriminalization laws by event time and overall summary of ATTs based on event-study/dynamic aggregation for yearly data across 48 US states with reliable estimates of microdosing trends

eTable 6. Dynamic effects of statewide recreational cannabis use laws by event time and overall summary of ATTs based on event-study/dynamic aggregation for yearly data across 48 US states with reliable estimates of microdosing trends

eTable 7. Dynamic effects of statewide medical cannabis use laws by event time and overall summary of ATTs based on event-study/dynamic aggregation for yearly data across 34 US states with reliable estimates of microdosing trends and no medical cannabis use laws as of 2010

This supplemental material has been provided by the authors to give readers additional information about their work.

© 2024 Yang KH et al. *JAMA Health Forum.*

eTable 1. Categorization of States with Medical or Recreational Cannabis Use Laws Enacted as of January 2024.

State	Medical	Enactment Date	Recreational	Enactment Date
Alabama	Yes	5/17/2021	No	
Alaska	Yes	11/3/1998	Yes	11/4/2014
Arizona	Yes	11/2/2010	Yes	11/3/2020
Arkansas	Yes	11/8/2016	No	
California	Yes	11/5/1996	Yes	11/8/2016
Colorado	Yes	11/7/2000	Yes	11/7/2012
Connecticut	Yes	6/1/2012	Yes	6/22/2021
Delaware	Yes	5/13/2011	Yes	4/23/2023
Florida	Yes	11/8/2016	No	
Georgia	No		No	
Hawaii	Yes	6/14/2000	No	
Idaho	No		No	
Illinois	Yes	8/1/2013	Yes	1/1/2020
Indiana	No		No	
Iowa	No		No	
Kansas	No		No	
Kentucky	No		No	
Louisiana	Yes	6/30/2015	No	
Maine	Yes	11/2/1999	Yes	11/8/2016
Maryland	Yes	6/1/2014	Yes	5/4/2023
Massachusetts	Yes	11/6/2012	Yes	12/15/2016
Michigan	Yes	11/4/2008	Yes	12/6/2018
Minnesota	Yes	5/29/2014	Yes	5/30/2023
Mississippi	Yes	2/2/2022	No	
Missouri	Yes	11/6/2018	Yes	12/8/2022
Montana	Yes	11/8/2016	Yes	11/3/2020

© 2024 Yang KH et al. *JAMA Health Forum.*

Nebraska	No		No	
Nevada	Yes	6/7/2001	Yes	1/1/2017
New Hampshire	Yes	7/23/2013	No	
New Jersey	Yes	1/18/2010	Yes	2/22/2021
New Mexico	Yes	04/2/2007	Yes	4/12/2021
New York	Yes	7/7/2014	Yes	3/31/2023
North Carolina	No		No	
North Dakota	Yes	11/8/2016	No	
Ohio	Yes	6/8/2016	No	
Oklahoma	Yes	6/26/2018	No	
Oregon	Yes	11/3/1998	Yes	11/4/2014
Pennsylvania	Yes	4/17/2016	No	
Rhode Island	Yes	1/3/2006	Yes	5/25/2022
South Carolina	No		No	
South Dakota	Yes	11/3/2020	No	
Tennessee	No		No	
Texas	No		No	
Utah	Yes	3/20/2018	No	
Vermont	Yes	5/19/2004	Yes	1/22/2018
Virginia	Yes	7/1/2020	Yes	2/5/2021
Washington	Yes	11/3/1998	Yes	11/6/2012
West Virginia	Yes	4/19/2017	No	
Wisconsin	No		No	
Wyoming	No		No	
Washington D.C.	Yes	1/1/2011	Yes	11/4/2014

© 2024 Yang KH et al. *JAMA Health Forum.*

eTable 2. Categorization of jurisdictions with psychedelic laws enacted as of January 2024.

Notes: ‘Local jurisdiction decriminalization’’ means there are cities or counties in the state that have decriminalized psychedelics

*Although Colorado legalized psychedelic use at the state level in 2022, Denver, which is within Colorado, decriminalized psychedelics on May 7, 2019.

† Although Washtenaw County decriminalized psychedelic use at the county level in 2021, Ann Arbor, which is within Washtenaw County, decriminalized psychedelics on September 21, 2020.

‡ Although Jefferson County decriminalized psychedelic use at the county level in 2023, Port Townsend, which is within Jefferson County, decriminalized psychedelics on December 20, 2021.

© 2024 Yang KH et al. *JAMA Health Forum.*

State	Jurisdiction	Legalization Status	Date Enacted	Reference
California	Arcata	Local jurisdiction decriminalization	10/06/2021	https://www.cityofarcata.org/DocumentCenter/View/11436/Resolution-212-17-Entheogenic-Plants-and-Fungi?bidId
California	Berkeley	Local jurisdiction decriminalization	07/11/2023	https://berkeleyca.gov/sites/default/files/documents/2023-07-11%20Item%2035%20Entheogenic_Psychedelic%20Hahn%20Sup.pdf
California	Oakland	Local jurisdiction decriminalization	05/09/2019	https://oakland.legistar.com/LegislationDetail.aspx?ID=3950933&GUID=5E53E7F6-F79F-433D-B669-0D687786590F&Options&Search
California	San Francisco	Local jurisdiction decriminalization	09/06/2022	https://sfgov.legistar.com/View.ashx?M=F&ID=11253021&GUID=3BE9445B-7D15-495D-A720-4713B724C43D
California	Santa Cruz	Local jurisdiction decriminalization	01/28/2020	https://ecm.cityofsantacruz.com/OnBaseAgendaOnline/Documents/V

© 2024 Yang KH et al. *JAMA Health Forum.*

				iewDocument/ENTHEOGENIC_PLANTS_RESOLUTION_-_REDLINE.DOCX?meetingId=1760&documentType=Agenda&itemId=17321&publishId=22851&isSection=false
Colorado*	Statewide	Legalization of psychedelic-assisted therapy & statewide psychedelic decriminalization	11/08/2022	https://www.sos.state.co.us/pubs/elections/Initiatives/titleBoard/filings/2021-2022/58Final.pdf
Colorado	Denver	Local jurisdiction decriminalization	05/07/2019	https://ballotpedia.org/Denver,_Colorado,_Initiated_Ordinance_301,_Psilocybin_Mushroom_Initiative_(May_2019)
District of Columbia	Washington DC	Local jurisdiction decriminalization	11/03/2020	https://ballotpedia.org/Washington,_D.C.,_Initiative_81,_Entheogenic_Plants_and_Fungus_Measure_(2020)
Maine	Portland	Local jurisdiction decriminalization	10/02/2023	https://www.psychedelicweek.com/p/portland-maine-council-decriminalize-psychedelic?utm_source=po

© 2024 Yang KH et al. *JAMA Health Forum.*

				st-email-title&publication_id=628557&post_id=137608711&utm_campaign=email-post-title&isFreemail=true&r=1ondlr&utm_medium=email
Massachusetts	Cambridge	Local jurisdiction decriminalization	02/03/2021	https://cambridgema.iqm2.com/Citizens/Detail_LegiFile.aspx?Frame=&MeetingID=2792&MediaPosition=&ID=13403&CssClass=
Massachusetts	Easthampton	Local jurisdiction decriminalization	10/20/2021	https://easthamptonma.gov/DocumentCenter/View/2806/2021---A-Resolution-Protecting-Adult-Access-to-Plant-Medicines--Prioritizing-Public-Health-Responses-to-Controlled-Substance-Possession-PDF
Massachusetts	Northampton	Local jurisdiction decriminalization	03/18/2021	https://northamptonma.gov/AgendaCenter/ViewFile/Item/16349?fileID=145419
Massachusetts	Salem	Local jurisdiction decriminalization	05/11/2023	https://www.salemnews.com/news/salem

© 2024 Yang KH et al. *JAMA Health Forum.*

				-leaders-scale-back-psilocybin-enforcement/article_c25ee422-f0f2-11ed-8917-c370b9fb56d5.html
Massachusetts	Somerville	Local jurisdiction decriminalization	01/14/2021	https://somervillecityma.iqm2.com/Citizens/Detail_LegiFile.aspx?Frame=&MeetingID=3289&MediaPosition=&ID=24035&CssClass=
Michigan	Detroit	Local jurisdiction decriminalization	11/02/2021	https://ballotpedia.org/Detroit,_Michigan,_Proposal_E,_Decriminalization_of_Entheogenic_Plants_Measure_(November_2021)
Michigan	Ferndale	Local jurisdiction decriminalization	02/27/2023	https://www.metrotimes.com/weed/ferndale-decriminalizes-magic-mushrooms-and-other-psychedelics-32494825
Michigan	Hazel Park	Local jurisdiction decriminalization	03/22/2022	https://www.clickondetroit.com/news/local/2022/03/22/hazel-park-decriminalizes-magic-mushrooms-

© 2024 Yang KH et al. *JAMA Health Forum.*

				psychedelic-plants/
Michigan	Washtenaw County†	Local jurisdiction decriminalization	11/12/2021	https://www.washtenaw.org/DocumentCenter/View/27212/Entheogenic-Plants-Policy
Michigan	Ann Arbor	Local jurisdiction decriminalization	09/21/2020	https://apnews.com/article/ann-arbor-plants-featured-ca-state-wire-mi-state-wire-b0ce69ca0961c150e0f900e8ea4cf432
Minnesota	Minneapolis	Local jurisdiction decriminalization	07/21/2023	https://www.minneapolismn.gov/government/mayor/executive-orders/executive-order-2023-01/
Oregon	Statewide	Legalization of psychedelic-assisted therapy & statewide psychedelic decriminalization	11/03/2020	https://sos.oregon.gov/admin/Documents/irr/2020/034text.pdf
Washington	Jefferson County‡	Local jurisdiction decriminalization	05/01/2023	https://www.marijuanamoment.net/washington-state-county-approves-psychedelics-decriminalization-resolution/
Washington	Port Townsend	Local jurisdiction decriminalization	12/20/2021	https://weblink.cityofpt.us/WebLink/DocView.aspx?id=203568&dbid=0&repo=PTDo

© 2024 Yang KH et al. *JAMA Health Forum.*

				cuments&cr=1
Washington	Seattle	Local jurisdiction decriminalization	10/04/2021	https://seattle.legistar.com/View.ashx?M=F&ID=9922100&GUID=9A8713B4-A6BD-4ABC-8E36-3151A175F003

eTable 3. Sensitivity analysis of cannabis and psychedelic policy effects on microdosing Google search trends changing the treatment period to 1 year prior to policy enactment.

Notes: 'Local psychedelic decriminalization laws' means there are cities or counties in the state that have decriminalized psychedelics;
Categorization of jurisdictions with medical cannabis use laws can be found in eTable 1 in the Supplement;
Categorization of jurisdictions with recreational cannabis use laws can be found in eTable 1 in the Supplement**;**
Categorization of jurisdictions with psychedelic decriminalization and assisted-therapy laws can be found in eTable 2 in the Supplement**;**

© 2024 Yang KH et al. *JAMA Health Forum.*

Excludes North Dakota, South Dakota, and Wyoming which had unreliable estimates throughout the period

Policy Change	Enactment Year	One Year Pre-Enactment	One Year Post-Enactment
	Effect Estimate (95% CI)	Effect Estimate (95% CI)	Effect Estimate (95% CI)
Local psychedelic decriminalization laws	22.4 (95% CI: 7.5 to 37.2)	22.6 (95% CI: 5.2 to 40.0)	19.7 (95% CI: 6.2 to 33.2)
Legalization of psychedelic-assisted therapy & statewide psychedelic decriminalization	28.9 (95% CI: 16.5 to 41.2)	37.7 (95% CI: 32.6 to 42.7)	26.5 (95% CI: 23.2 to 29.8)
Statewide recreational cannabis use laws	40.9 (95% CI: 28.6 to 53.3)	39.9 (95% CI: 27.3 to 52.6)	44.6 (95% CI: 31.2 to 58.0)
Statewide medical cannabis use laws	11.5 (95% CI: 6.0 to 16.9)	9.2 (95% CI: 4.2 to 14.2)	11.1 (95% CI: 4.1 to 18.1)

eTable 4. Dynamic effects of local jurisdiction psychedelic decriminalization laws by event time and overall summary of ATTs based on event-study/dynamic aggregation for yearly data across 48 US states with reliable estimates of microdosing trends.

Notes: ATT = Average treatment effect on the treated; Overall = Overall summary of ATTs based on event-study/dynamic aggregation
Control Group: Never Treated, Anticipation Periods: 0
Estimation Method: Doubly Robust

Event Time	Effect Estimate (95% CI)
overall	22.4 (95% CI: 7.5 to 37.2)

© 2024 Yang KH et al. *JAMA Health Forum.*

-12	0.3 (95% CI: -1.3 to 1.9)
-11	1.5 (95% CI: -0.7 to 3.6)
-10	-0.2 (95% CI: -1.5 to 1.1)
-9	0.9 (95% CI: -1.6 to 3.5)
-8	1.0 (95% CI: -0.6 to 2.6)
-7	-0.8 (95% CI: -3.0 to 1.3)
-6	3.2 (95% CI: -1.9 to 8.4)
-5	-2.8 (95% CI: -9.8 to 4.2)
-4	4.8 (95% CI: -3.7 to 13.3)
-3	7.2 (95% CI: 2.2 to 12.1)
-2	2.4 (95% CI: -5.3 to 10.1)
-1	3.0 (95% CI: -4.4 to 10.5)
0	5.2 (95% CI: 1.3 to 9.1)
1	13.5 (95% CI: -2.1 to 29.1)
2	16.3 (95% CI: 4.7 to 28.0)
3	27.3 (95% CI: -1.1 to 55.7)
4	49.5 (95% CI: -17.4 to 116.3)

eTable 5. Dynamic effects of legalization of psychedelic-assisted therapy & statewide psychedelic decriminalization laws by event time and overall summary of ATTs based on event-study/dynamic aggregation for yearly data across 48 US states with reliable estimates of microdosing trends.

Notes: ATT = Average treatment effect on the treated; Overall = Overall summary of ATTs based on event-study/dynamic aggregation
Control Group: Never Treated, Anticipation Periods: 0
Estimation Method: Doubly Robust

Event Time	Effect Estimate (95% CI)
overall	28.9 (95% CI: 16.5 to 41.2)

© 2024 Yang KH et al. *JAMA Health Forum.*

-11	0.2 (95% CI: -1.1 to 1.5)
-10	0.2 (95% CI: -1.2 to 1.7)
-9	3.1 (95% CI: -2.3 to 8.6)
-8	-2.6 (95% CI: -10.3 to 5.2)
-7	-0.1 (95% CI: -1.7 to 1.6)
-6	3.9 (95% CI: 0.4 to 7.5)
-5	10.3 (95% CI: -0.9 to 21.5)
-4	9.7 (95% CI: -6.6 to 26.0)
-3	19.6 (95% CI: 4.5 to 34.7)
-2	7.8 (95% CI: 5.5 to 10.2)
-1	12.1 (95% CI: 0.0 to 24.2)
0	16.3 (95% CI: -10.5 to 43.2)
1	30.9 (95% CI: 2.8 to 59.1)
2	29.3 (95% CI: 21.6 to 36.9)
3	38.9 (95% CI: 32.6 to 45.3)

eTable 6. Dynamic effects of statewide recreational cannabis use laws by event time and overall summary of ATTs based on event-study/dynamic aggregation for yearly data across 48 US states with reliable estimates of microdosing trends.

Notes: ATT = Average treatment effect on the treated; Overall = Overall summary of ATTs based on event-study/dynamic aggregation
Control Group: Never Treated, Anticipation Periods: 0
Estimation Method: Doubly Robust

Event Time	Effect Estimate (95% CI)
overall	40.9 (95% CI: 28.6 to 53.3)
-12	0.5 (95% CI: -1.9 to 2.9)
-11	3.8 (95% CI: -3.9 to 11.5)

© 2024 Yang KH et al. *JAMA Health Forum.*

-10	-0.5 (95% CI: -5.9 to 4.9)
-9	1.4 (95% CI: -2.9 to 5.7)
-8	0.1 (95% CI: -1.1 to 1.4)
-7	0.2 (95% CI: -2.9 to 3.4)
-6	1.3 (95% CI: -1.0 to 3.6)
-5	-1.1 (95% CI: -4.0 to 1.9)
-4	1.6 (95% CI: -3.0 to 6.2)
-3	4.0 (95% CI: -1.6 to 9.5)
-2	-0.0 (95% CI: -4.2 to 4.2)
-1	2.7 (95% CI: -0.9 to 6.2)
0	1.2 (95% CI: -2.9 to 5.2)
1	8.3 (95% CI: 3.0 to 13.6)
2	12.3 (95% CI: 1.5 to 23.1)
3	21.7 (95% CI: 7.3 to 36.1)
4	29.6 (95% CI: 8.5 to 50.6)
5	32.4 (95% CI: 17.0 to 47.7)
6	41.0 (95% CI: 23.5 to 58.5)
7	51.3 (95% CI: 31.3 to 71.2)
8	44.2 (95% CI: -2.9 to 91.4)
9	61.1 (95% CI: 20.6 to 101.5)
10	89.3 (95% CI: 48.1 to 130.4)
11	99.0 (95% CI: 70.2 to 127.7)

© 2024 Yang KH et al. *JAMA Health Forum.*

eTable 7. Dynamic effects of statewide medical cannabis use laws by event time and overall summary of ATTs based on event-study/dynamic aggregation for yearly data across 34 US states with reliable estimates of microdosing trends and no medical cannabis use laws as of 2010.

Notes: ATT = Average treatment effect on the treated; Overall = Overall summary of ATTs based on event-study/dynamic aggregation
Control Group: Never Treated, Anticipation Periods: 0
Estimation Method: Doubly Robust

Event Time	Effect Estimate (95% CI)
overall	11.5 (95% CI: 6.0 to 16.9)
-11	-1.3 (95% CI: -4.6 to 1.9)
-10	-3.0 (95% CI: -9.1 to 3.1)
-9	0.9 (95% CI: -3.8 to 5.5)
-8	-0.5 (95% CI: -4.1 to 3.1)
-7	2.3 (95% CI: -3.6 to 8.1)
-6	-1.1 (95% CI: -5.3 to 3.2)
-5	0.0 (95% CI: -2.1 to 2.2)
-4	-0.2 (95% CI: -3.3 to 2.8)
-3	0.5 (95% CI: -2.1 to 3.1)
-2	0.3 (95% CI: -3.0 to 3.6)
-1	1.2 (95% CI: -1.3 to 3.7)
0	0.3 (95% CI: -2.9 to 3.6)
1	1.8 (95% CI: -1.6 to 5.1)
2	1.1 (95% CI: -3.3 to 5.6)
3	5.3 (95% CI: 0.5 to 10.1)
4	3.8 (95% CI: -3.2 to 10.9)
5	6.8 (95% CI: -1.0 to 14.6)
6	8.2 (95% CI: -1.3 to 17.7)
7	7.9 (95% CI: -3.5 to 19.4)
8	10.4 (95% CI: 1.0 to 19.8)
9	14.3 (95% CI: -3.1 to 31.7)
10	22.8 (95% CI: 5.2 to 40.3)
11	24.7 (95% CI: 12.2 to 37.1)
12	41.6 (95% CI: 17.9 to 65.2)

© 2024 Yang KH et al. *JAMA Health Forum.*

© 2024 Yang KH et al. *JAMA Health Forum.*
