# Supplementary material for: State Cannabis and Psychedelic Legislation and Microdosing Interest in the US
Source: JAMA Health Forum. 2024 Jun 28;5(6):e241653. doi: 10.1001/jamahealthforum.2024.1653 (PMC11214114; doi:10.1001/jamahealthforum.2024.1653)
Supplement: Supplement 2. — Data Sharing Statement [file jamahealthforum-e241653-s002.pdf]

Data Sharing Statement

Yang. State Cannabis and Psychedelic Legislation and Microdosing Interest in the US. *JAMA Health Forum*. Published June 28, 2024. doi:10.1001/jamahealthforum.2024.1653

Data
Data available: Yes
Data types: Data (not involving human participants)
How to access data: All data used in this analysis are publicly available. Additional information on data analysis is available from the authors upon request.
When available: With publication

Supporting Documents
Document types: None

Additional Information
Who can access the data: Anyone requesting the data
Types of analyses: For any purpose
Mechanisms of data availability: With investigator support
